# Plasmatic Soluble Receptor for Advanced Glycation End Products as a New Oxidative Stress Biomarker in Patients with Prosthetic-Joint-Associated Infections?

**DOI:** 10.1155/2017/6140896

**Published:** 2017-12-13

**Authors:** Luca Massaccesi, Barbara Bonomelli, Monica Gioia Marazzi, Lorenzo Drago, Massimiliano Marco Corsi Romanelli, Daniela Erba, Nadia Papini, Alessandra Barassi, Giancarlo Goi, Emanuela Galliera

**Affiliations:** ^1^Department of Biomedical, Surgical and Dental Sciences, Università degli Studi di Milano, Milan, Italy; ^2^Department of Biomedical Sciences for Health, Università degli Studi di Milano, Milan, Italy; ^3^IRCCS Galeazzi Orthopaedic Institute, Milan, Italy; ^4^U.O.C SMEL-1 Patologia Clinica IRCCS Policlinico San Donato, San Donato, Milan, Italy; ^5^Department of Food, Environmental and Nutritional Sciences (DeFENS), Università degli Studi di Milano, Milan, Italy; ^6^Department of Medical Biotechnology and Translational Medicine, Università degli Studi di Milano, Milan, Italy; ^7^Department of Health Sciences, Università degli Studi di Milano, Milan, Italy

## Abstract

Prosthetic joint infection (PJI) is the most common cause of failure of total joint arthroplasty, but a gold standard for PJI diagnosis is still lacking. Advanced glycation end products (AGEs) are proinflammatory molecules inducing intracellular oxidative stress (OS) after binding to their cell membrane receptors (RAGE). The aim of this study was to evaluate plasmatic soluble receptor for advanced glycation end products (sRAGE), as a new OS and infection marker correlating sRAGE to the level of OS and antioxidant defenses, in PJI, in order to explore the possible application of this new biomarker in the early diagnosis of PJI. Plasmatic sRAGE levels (by ELISA assay), plasma antioxidant total defenses (by lag time method), plasma reactive oxygen species (ROS), and thiobarbituric acid reactive substance (TBARS) levels (by colorimetric assay) were evaluated in 11 PJI patients and in 30 matched controls. ROS and TBARS were significantly higher (*p* < 0.001) while plasma total antioxidant capacity and sRAGE were significantly lower (*p* < 0.01) in patients with PJI compared to controls. Our results confirm the OS in PJI and show a strong negative correlation between the level of sRAGE and oxidative status, suggesting the plasmatic sRAGE as a potential marker for improving PJI early diagnosis.

## 1. Introduction

The numbers of primary total hip and total knee arthroplasties have been increasing over the past decade. Prosthetic joints improve the quality of life, but they may fail, thus requiring revision surgery. Infection is the most serious complication, occurring in 0.8 to 1.9% of knee arthroplasties and 0.3 to 1.7% of hip arthroplasties. Nowadays, a wide number of tests are available for prosthetic-joint-associated infection (PIJ) diagnosis, ranging from haematological markers of infection and inflammation, intraoperative culture, and histology analysis. Nevertheless, there is still a lack of gold standards for the diagnosis of PIJ [1, 2] because the clinical presentation of PJI is often ambiguous and classical inflammatory markers can be misleading [3, 4]. In order to optimize the diagnostic process, infection biomarkers with fast response and high sensitivity and specificity for infection are needed [5–8].

Among the scenario of infection diagnosis, an emerging role has been recently described for oxidative stress (OS) evaluation [9, 10]. Inflammatory response induces an overproduction of ROS, exacerbating organ and tissue injuries [11]. PJI is mostly due to *Staphilococcus aureus* infection, which induces massive leukocyte recruitment, mainly neutrophils, to the site of infection. Neutrophils represent the first line defense against pathogen invasion, and, in order to combat *S. aureus,* neutrophils are able both to engulf bacteria through phagocytosis and to directly kill the bacteria by ROS production, leading to oxidative burst [12]. Oxidative burst is a major event characterizing the inflammatory response, and for this reason, markers of oxidative stress are currently used in the clinical evaluation of sepsis [11, 13], but at the moment no evidence has described so far the use of OS stress evaluation in PJI diagnosis.

Recent evidences correlated OS to the levels of advanced glycation end products (AGEs): In particular, oxidative stress has been recently described as able to increase the formation of AGEs [14, 15]. AGEs are a heterogeneous group of irreversible adduct formed by the nonenzymatic glycation and glycoxidation of proteins, nucleic acids, and lipids. AGEs interact with their receptors RAGE, which exist as a membrane-bound form and a soluble plasmatic form, soluble receptor for advanced glycation end products (sRAGE). Interaction of AGEs with the cell membrane-bound receptor RAGE activates NF-*κ*B, increases gene expression and production of inflammatory cytokines, and increases the production of ROS [16]. On the contrary, sRAGE act as a decoy receptor counteracting the action of RAGE by competing for AGE ligand, thus having a protective role against deleterious effects mediated by AGE-RAGE axis [15]. Soluble RAGE have been recently proposed as a circulating biomarker indicating the status of disease [17] and oxidative stress [18, 19].

The aim of the present study was to evaluate the diagnostic value of plasmatic sRAGE, as new oxidative stress and infection marker, correlating it to the level of OS and antioxidant defenses, in postoperative prosthetic joint infection (PJI), in order to explore the possible application of this new biomarker in the early diagnosis of PJI.

## 2. Materials and Methods

### 2.1. Subjects

Eleven patients with prosthetic-joint-associated infection (PJI), aged 60.2 ± 16, were recruited from C.R.I.O. Unit, IRCCS Galeazzi, Milan, Italy. Controls were 30 adult volunteer blood donors, aged 56.1 ± 19.41, from the Italian association of blood volunteers (AVIS) in Milan, Italy. This investigation conforms to the principles outlined in the Declaration of Helsinki. Signed informed written consent was obtained from all subjects before their participation in the study. The manuscript is part of a larger project on the search of new biomarkers for PJI diagnosis (“*Ricerca Corrente note L4061*”), approved by the local ethical committee.

### 2.2. Materials

Commercial chemicals were of the highest available grade. The water routinely used was freshly redistilled in a glass apparatus. Bovine serum albumin (BSA), copper(II) sulphate (CuSO_4_), 2-thiobarbituric acid (TBA), butylated hydroxyl toluene (BHT), and 1,1,3,3-tetramethoxypropane (TMP) were purchased from Sigma Chemical Co. (St. Louis, MO, USA). All other reagents were purchased from Merck (Darmstad, Germany). d-ROMs kit test was purchased from Diacron International (Grosseto, Italy).

### 2.3. Blood Samples and Serum/Plasma Preparation

Plasma was prepared from heparinized venous blood. After collection, blood samples were immediately centrifuged for 15 min at 3000 ×g and plasma immediately withdrawn and stored at −70°C until ELISA assay and evaluation of plasmatic oxidative status.

### 2.4. Evaluation of Plasma Oxidative

Plasma lipid hydroperoxide levels (ROS) were determined colorimetrically according to Trotti et al. [20] and expressed as H_2_O_2_ equivalents.

Thiobarbituric acid reactive substances (TBARS) in plasma samples were measured spectrophotometrically. Briefly, the sample was mixed with 10 mmol/L BHT (in absolute ethanol), 1% orthophosphoric acid (in HCl 0.1 N), and 0.6% TBA and then heated at 90°C for 45 min. After cooling, the pink MDA-(TBA)_2_ adduct was extracted in n-butanol and the absorbance of the organic phase was determined at 535 nm. The absorbance of blank (plasma without TBA reactive) was subtracted from that of the corresponding sample. TMP was used as standard of MDA, whose concentrations in samples were calculated with respect to a standard curve (range 1–10 *μ*mol/L).

The kinetics of plasma oxidation, induced by addition of CuSO_4_ 0.5 M, were determined at 37°C by monitoring the development of fluorescence at 430 nm, setting the excitation at 355 nm as described by Cervato et al. [21] by Multilabel Counter Wallac 1420 from PerkinElmer. This method allows the evaluation of the peroxidation kinetics monitored following the formation of fluorescent adducts originating from the reaction of aldehydes (derived from lipid peroxidation promoted by Cu^++^ bound to apolipoproteins) with amino groups of plasma proteins and/or phospholipids. The kinetic is expressed by a sigmoid curve that can be divided into an initial latency phase, followed by a second propagation phase. The initial latency phase (lag time, expressed in minutes and calculated as the intercept of the linear regression of the propagation phase with that of the latency phase) is an index of lipoprotein resistance to peroxidation.

### 2.5. sRAGE ELISA Assay

Levels of soluble RAGE in plasma were determined by ELISA commercial assays, according to the manufacturers' instructions (sRAGE: R&D Systems, Minneapolis, Minnesota, USA).

For the sRAGE assay, the sensitivity was 4.44 pg/mL, and intra- and interassay coefficients of variation were 2.4% and 4.7%, respectively.

### 2.6. Statistical Analysis

The Shapiro–Wilk test showed no significative difference from normal distribution. Therefore, parametric techniques were used. Means were compared by Student *t*-test. The Pearson correlation coefficient (*r*^2^) was calculated to determine the correlation between values measured by different assays. Distribution and correlation analysis were performed using the SPSS STATISTIC 24 package (SPSS Inc., Chicago, IL, USA).

Statistical analysis of receiver operating characteristic (ROC) curves and area under the curve (AUC) was performed using Prism 5 software.

## 3. Results

### 3.1. Plasma Peroxidation Parameters

**Figure 1:**
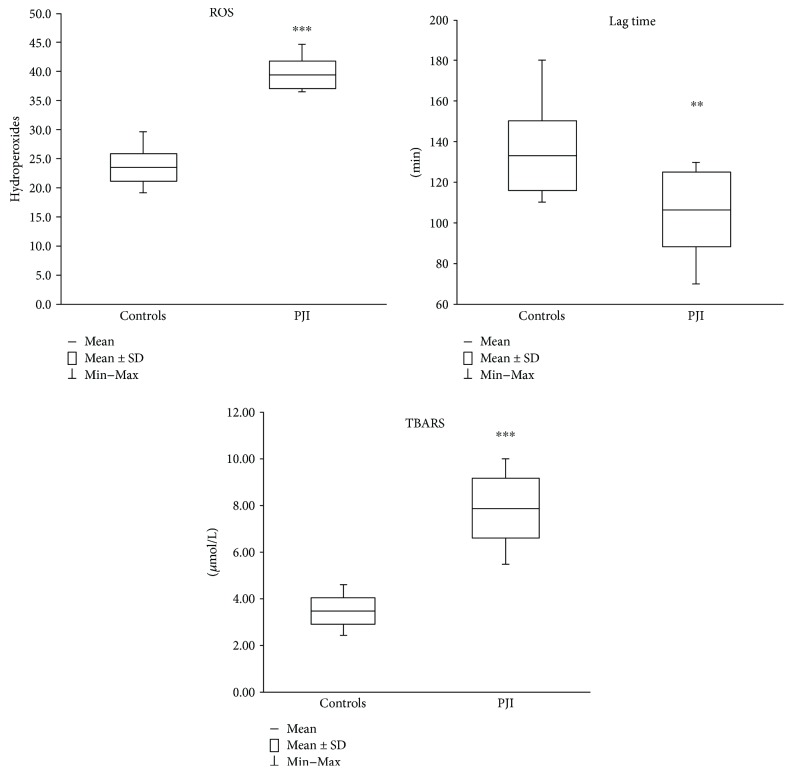
Plasma peroxidation parameters. Hydroperoxides are expressed as equivalent of H_2_O_2_ mg/dL of plasma. Values are expressed as mean ± standard deviation (SD). ^∗∗^*p* < 0.01 controls versus PJI subjects. ^∗∗∗^*p* < 0.001 controls versus PJI subjects.

Plasma peroxidation parameters are reported in Figure 1. As expected, hydroperoxide levels (Figure 1(a)) of PJI patients (mean ± SD 40.4 ± 2.4 mg/dL; range 37.4–45.7 mg/dL) are significantly (*p* < 0.001) higher (+72%) than the controls (mean ± SD 23.51 ± 2.52 mg/dL; range 19.17–29.65 mg/dL); the time interval necessary for inhibiting the Cu-induced peroxidative process (lag time, Figure 1(b)) is significantly (*p* < 0.01) lower (−20%) in PJI patients (107 ± 18 min; 70–130 min) than in the control group (133 ± 17 min; 110–180 min), and TBARS levels (Figure 1(c)) are significantly (*p* < 0.001) higher (+132%) in PJI patients (8.03 ± 1.31 *μ*mol/L; 5.60–10.20 *μ*mol/L) with respect to the control group (3.46 ± 0.61 *μ*mol/L; 2.42–4.61 *μ*mol/L).

### 3.2. sRAGE

**Figure 2:**
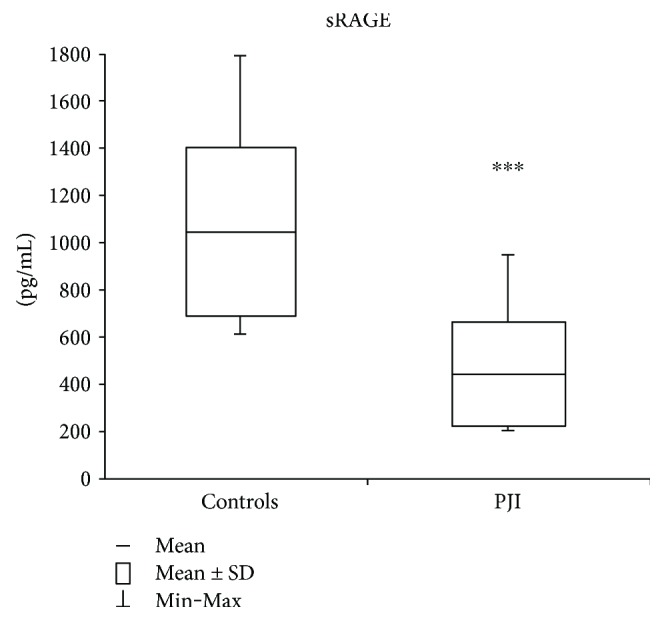
sRAGE plasmatic levels. Values are expressed as mean ± standard deviation (SD). ^∗∗∗^*p* < 0.001.

As shown in Figure 2, sRAGE levels are significantly (*p* < 0.001) lower (−58%) in PJI subjects (442.96 ± 221.21 pg/mL; 202, 81–950, 39 pg/mL) than in the control group (1044.84 ± 359.13 pg/mL; 609.53–1794.03 pg/mL).

### 3.3. Correlation Analysis

**Figure 3:**
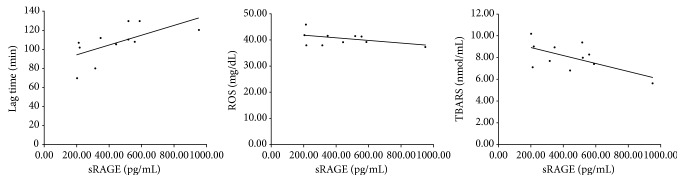
Correlation analysis between sRAGE levels and lag time (*r*^2^ = 0.614), ROS (*r*^2^ = −0.434 not significant), and TBARS (*r*^2^ = −0.609).

As shown in Figure 3, in PJI patients, sRAGE show a linear significant (*p* < 0.05) positive correlation (*r*^2^ = 0.614) with lag time and a linear significant (*p* < 0.05) negative correlation (*r*^2^ = −0.609) with TBARS levels, as well as a linear negative (even not significant) correlation with ROS levels, suggesting this parameter as a good/useful marker of oxidative status/stress.

### 3.4. ROC Curve Analysis

In order to evaluate the diagnostic potential of the biomarker analyzed in the detection of PJI, ROC curve analysis was performed for all four parameters and AUC was calculated.

**Figure 4:**
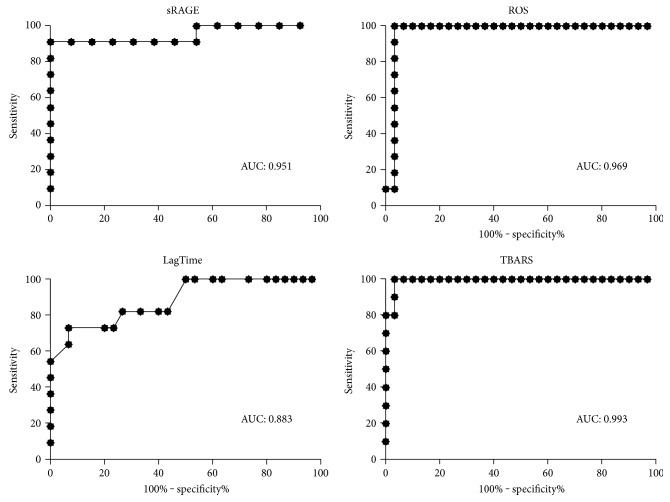
Receiver operating characteristics (ROC) curve and area under the curve (AUC).

As shown in Figure 4, each parameter displayed an AUC higher than the cut-off 0.8 considered the threshold of AUC to consider a parameter clinically acceptable. The AUC resulted 0.951, 0.969, 0.883, and 0.993 for sRAGE, ROS, lag time, and TBARS, respectively.

## 4. Discussion

The numbers of primary total hip and total knee arthroplasties increased over the past decade, but the complications due to defective implants and prosthetic infection are still challenging.

Several studies have underlined the crucial role of oxidative stress in the pathophysiologic “vicious cycle” of inflammation, deeply related to infection [22].

ROS are normally generated by cellular metabolism and at low or moderate concentrations play physiological roles including cellular response to infectious agents. Oxidative stress occurs when the production of ROS and other reactive species overwhelm the capacity of cellular antioxidant defenses to detoxify these potentially injurious species. Redox imbalance can be produced through an increased generation of ROS and by the decrease of cellular antioxidant molecules. Oxidative stress has been implicated in pathological conditions associated with different human inflammatory diseases. Excessive ROS have been linked to pathogenesis of cancer, cardiovascular disease, atherosclerosis, hypertension, ischemia/reperfusion injury, diabetes mellitus, neurodegenerative diseases, rheumatoid arthritis, pulmonary disease, and ageing [23–27]. Increasing data suggest that oxidative stress is also involved in the pathogenesis of infectious diseases [28]. Among the factors regulating oxidative stress, an important role is played by the axis AGE-RAGE. On the one hand, AGE-RAGE axis activated NADPH oxidase; on the other hand, it induces the formation of reactive oxygen species (ROS), which induce cellular damage acting as cellular toxicant. This effect is defined as AGE-RAGE-oxidative stress (AROS) [29], and it is involved in the pathogenesis of different inflammatory diseases. The function of AGE-RAGE axis is counterbalanced by the soluble receptor sRAGE, which act as a decoy for AGE ligands, exerting a protective role against tissue damages induced by AROS. Being a circulating molecule, sRAGE have been investigated as potential biomarker of oxidative stress [30, 31] and disease status [17], but evidences about the diagnostic role of sRAGE as marker of oxidative stress and PJI are still lacking.

On these bases, the study was aimed to evaluate the diagnostic value of plasmatic sRAGE by correlating it to the plasmatic levels of OS and antioxidant defenses in postoperative prosthetic joint infection patients (PJI), in order to explore the possible application of this new biomarker in the early diagnosis of PJI.

The oxidative stress (OS) has been evaluated using classical methodologies (plasma hydroperoxide levels and thiobarbituric acid reactive substances, *TBARS*) and measuring total plasma antioxidant capacity by lag time method, a simple and reproducible assay used to evaluate the “oxidation resistance” of plasma lipoproteins. This analytical approach has been successfully applied for monitoring plasmatic peroxidation risk in patients with oxidative-related pathologies (such as, diabetes, cancer, hypertension, Down syndrome, chronic renal failure, etc.) [32–34] as well as to evaluate the effects of antioxidant treatments.

The evaluation of both markers of peroxidation and plasma oxidative defenses (Figure 1) highlights a condition of strong oxidative stress in PJI as occurring in other pathologies [33].

Consistently with these results, sRAGE resulted significantly lower in PJI patients, confirming its protective role as decoy receptor for AGE ligand.

This role seems to be also confirmed by the correlation analysis, which indicated a negative correlation of sRAGE with ROS, and TBARS levels, and a positive correlation of sRAGE with lag time. The oxidative stress increase observed in PJI, as indicated by the analyzed OS parameters, corresponds to a decrease of lipoprotein resistance to peroxidation, measured by lag time, and it consistently correlates with the decrease of sRAGE levels.

The diagnostic role of circulating low levels of sRAGE is controversial. Serum levels of sRAGE are lower in healthy subjects than in patients with coronary artery disease and atherosclerotic burden disease in nondiabetic subjects [35–37]. Low levels of sRAGE have also been described in hypercholesterolemia [38], essential hypertension [39], Alzheimer disease, and vascular dementia.

On the other hand, other evidences described elevated circulating sRAGE in type 2 diabetic patients with coronary artery disease or with atherosclerotic burden. The reason for this elevated levels of sRAGE could be due to elevated levels of matrix metalloproteinases (MMPs) in diabetes and renal disease, which would increase the formation of sRAGE [17].

PJI induces an inflammatory response involving cytokine production and oxidative stress; therefore, the reduction of the decoy receptor sRAGE in PJI patients compared to the control group is consistent with a loss of sRAGE protective role.

The diagnostic value of sRAGE and oxidative stress biomarkers was further evaluated by the analysis of receiver operating characteristics (ROC) curve and AUC (area under the curve).

The closer the curve was to the upper left-hand corner of the SROC curve plot, the better the overall accuracy of the test. An area under the ROC curve between 0.90 and 1.0 is considered as excellent diagnostic accuracy, between 0.80 and 0.90 as good, between 0.70 and 0.80 as fair, between 0.60 and 0.70 as poor, and between 0.50 and 0.60 as fail [40].

SRAGE displayed a good AUC (0.951), indicating that this molecule has a good diagnostic value and it could be considered as a new biomarker for the diagnosis of PJI. Similarly, the parameters evaluating the oxidative status, ROS, lag time, and TBARS displayed good AUC values (0.969, 0.833, and 0.933, resp.), far over the value of 0.8 which is considered in the clinical practice the lower cut-off for a parameter to be considered clinically acceptable. These results on the one hand indicate the good diagnostic tool of the oxidative biomarkers in the detection of PJI and on the other hand indicate that sRAGE can be considered a good marker of oxidative status and, therefore, also a good biomarker of PJI.

## 5. Conclusion

Our results confirm the substantial OS in PJI and show a strong negative correlation between the level of sRAGE and oxidative stress, suggesting that plasmatic sRAGE can be considered as potential OS markers. This new approach could represent a useful diagnostic tool for improving prosthesis joint infection diagnosis, where a clear detection of the infection is still lacking, in addition to routine inflammatory parameters.
